# Advanced analytical methods to assess physical activity behaviour using accelerometer raw time series data: a protocol for a scoping review

**DOI:** 10.1186/s13643-020-01515-2

**Published:** 2020-11-07

**Authors:** Tripti Rastogi, Anne Backes, Susanne Schmitz, Guy Fagherazzi, Vincent van Hees, Laurent Malisoux

**Affiliations:** 1 Physical Activity, Sport and Health Research Group, Luxembourg Institute of Health, 76 rue d’Eich, L-1460 Luxembourg, Grand Duchy of Luxembourg; 2 Competence Center for Methodology and Statistics, Luxembourg Institute of Health, 1A-B rue Thomas Edison, L-1445 Strassen, Grand Duchy of Luxembourg; 3 Digital Epidemiology Hub, Luxembourg Institute of Health, 1A-B rue Thomas Edison, L-1445 Strassen, Grand Duchy of Luxembourg; 4 Netherlands eScience Center, Science Park 140 (Matrix I), 1098 XG Amsterdam, The Netherlands; 5 Amsterdam UMC, Department of Public and Occupational Health, Amsterdam Public Health Research Institute, Vrije Universiteit Amsterdam, Amsterdam, The Netherlands; 6 Accelting, Almere, The Netherlands

**Keywords:** Tri-axial accelerometers, Wearables, Sensors, Data processing, Algorithm, Physical activity pattern

## Abstract

**Background:**

Physical activity (PA) is a complex multidimensional human behaviour. Currently, there is no standardised approach for measuring PA using wearable accelerometers in health research. The total volume of PA is an important variable because it includes the frequency, intensity and duration of activity bouts, but it reduces them down to a single summary variable. Therefore, analytical approaches using accelerometer raw time series data taking into account the way PA are accumulated over time may provide more clinically relevant features of physical behaviour. Advances on these fields are highly needed in the context of the rapid development of digital health studies using connected trackers and smartwatches. The objective of this review will be to map advanced analytical approaches and their multidimensional summary variables used to provide a comprehensive picture of PA behaviour.

**Methods:**

This scoping review will be guided by the Arksey and O’Malley methodological framework. A search for relevant publications will be undertaken in MEDLINE (PubMed), Embase and Web of Science databases. The selection of articles will be limited to studies published in English from January 2010 onwards. Studies including analytical methods that go beyond total PA volume, average daily acceleration and the conventional cut-point approaches, involving tri-axial accelerometer data will be included. Two reviewers will independently screen all citations, full-text articles and extract data. The data will be collated, stored and charted to provide a descriptive summary of the analytical methods and outputs, their strengths and limitations and their association with different health outcomes.

**Discussion:**

This protocol describes a systematic method to identify, map and synthesise advanced analytical approaches and their multidimensional summary variables used to investigate PA behaviour and identify potentially clinically relevant features. The results of this review will be useful to guide future research related to analysing PA patterns, investigate their association with health conditions and suggest appropriate recommendations for changes in PA behaviour. The results may be of interest to sports scientists, clinical researchers, epidemiologists and smartphone application developers in the field of PA assessment.

**Scoping review registration:**

This protocol has been registered with the Open Science Framework (OSF): https://osf.io/yxgmb.

## Background

Physical activity (PA) plays a key role in health and well-being of an individual. PA is beneficial for all age groups and has both short- and long-term benefits [1]. There is strong evidence that PA reduces the risk of cancer and cardiovascular mortality [1, 2]. Also, PA prevents several lifestyle disorders such as diabetes, cardiovascular diseases, arterial stiffness, obesity and metabolic syndrome [3–5]. In addition, higher amounts of PA and/or lesser sedentary behaviour are associated with a lesser risk of depression [6] and sleep disorders [7].

In the era of digital development, the use of smartphone applications and other digital devices like wearable PA monitors has become popular for tracking PA in the free-living environment. Recent developments in wearable technology enable regular and long-term tracking of PA, representing a promising measure for the promotion and support of a more active lifestyle [8]. These devices provide feedback in form of step counts, energy expenditure or total time spent in moderate to vigorous intensity. This feedback can encourage behavioural change towards a more active and less sedentary lifestyle [9]. Indeed, several studies demonstrated that PA monitors and smartphone applications can be used to promote PA in individuals [10, 11].

Nowadays, there is an increasing amount of clinical and population-based observational studies evaluating the amount of PA of a person using wearable devices such as smartwatches, smartphones, consumer-grade or research-grade activity monitors. A common characteristic of these devices is that they include an accelerometer (a Micro Electro-Mechanical System or an Inertial Measurement Unit which consists of an accelerometer, a gyroscope and a magnetometer) which measures acceleration relative to the Earth’s gravitational field. Compared to questionnaire-based assessments, wearable devices present the advantage of detailed and objective measurements of the PA behaviour in the free-living environment [12]. Especially when the PA duration or the intensity is of interest, self-reported results should be interpreted with caution [12]. Whilst the questionnaires provide information on the purpose of the activity (and sometimes on activity type), the wearable devices quantify the motion performed. Thus, both approaches provide complementary information, which are not interchangeable [13].

The output of the accelerometers is a three-dimensional time series of accelerations expressed in gravitational units. Developments in the field of computing techniques for the domain of health sciences have made analytical tools more easily available [14], and enabled researchers from both academic and industrial milieu to decipher the 24-h raw time series acceleration signal. These analytical tools can now generate a number of summary variables. However, until now, most studies are limited to measuring PA in units of time spent in certain intensity levels, which can subsequently be used to compare sub-groups by categorising people as active or inactive [15, 16]. The latter classification is usually based on PA recommendations of 150 min of moderate PA per week or 75 min of vigorous PA or an equivalent combination of both [17], although many alternative recommendations exist [18]. The classification approach is practical and convenient but does not reveal a complete PA profile of a person. Actually, cut-point approaches comes with several limitations such as (1) the complex relationships of acceleration with energy expenditure, activity types and study populations, (2) the many parameters that are somehow arbitrarily determined (e.g. bout length), and (3) the collinearity between PA intensity categories [19]. The latter implies that the time spent in different PA intensity categories cannot be used in standard regression models because of the compositional nature of the data [5].

How a person accumulates minutes of sedentary time was demonstrated to be a key determinant regulating different biomarkers of obesity [20]. Similarly, how a person accumulates active time is of significant importance to provide adequate guidance and appropriate recommendation for each individual [18]. For example, some people may be moderate to vigorously active for a long bout of exercise whilst spending the rest of the day in sedentary behaviour, whereas some others may be active for frequent short durations by doing household work, gardening, commuting from home to office, etc. Each type of PA behaviour pattern has different health implications [21]. Indeed, PA is a complex multidimensional human behaviour, which dimensions include amongst others frequency (e.g. number of light activities per week), intensity (e.g. exercise or active sports are usually in moderate to vigorous intensities), time (e.g. time spent sitting), type (e.g. walking) or posture (e.g. sitting), each describing a different aspect of the PA behaviour. The total volume of PA (e.g. total time spent at light intensity) represents an important variable as it combines the intensity and time dimensions of PA. However, it reduces both dimensions to a single summary variable, which comes with a loss of information. In other words, the way PA is accumulated over time does not influence the results. Therefore, advanced approaches taking into account the way PA is accumulated over time and providing multidimensional output may be more suitable to analyse different patterns of activity, investigate their association with health conditions and suggest appropriate recommendations for changes in PA behaviour [18].

Over the last few years, more advanced analytical approaches to generate new summary variables capturing more appropriately the multidimensional nature of PA behaviour have emerged [22–24]. Irrespective of whether these advanced analytical approaches are cut-point dependent or independent, they allow generating a comprehensive profile of PA pattern (Fig. 2). Accelerometer time-series raw data presents the possibility to evaluate and test whether detailed patterns of activity may be more informative to health outcomes than traditional measures of total activity.

In fact, accelerometry is a field in health sciences that emerged in the 1990s and is still very actively exploring analytical techniques. Currently, there is no universally accepted and standardised approach for measuring PA using wearable accelerometers in health research. Thus, there is considerable heterogeneity in methodology, data acquisition and data processing. Several published systematic reviews aimed to analyse the methods and results of calibration studies relative to energy expenditure (i.e. PA intensity) [25], data processing criteria applied to the acceleration signal [26] and the completeness of accelerometer reporting methods [27]. A preliminary search revealed that none of the previous reviews have mapped existing multidimensional PA summary variables for assessing PA and investigating the association with health outcomes.

The choice of PA summary variables must be based on its ability to reveal clinically relevant features of physical behaviour and to discriminate between experimental groups. Previous authors have supported the need to overcome the limitation of unidimensional PA related-outcomes in defining the activity profile of a person [21, 28]. Some studies have incorporated a variety of equally important characteristics of PA, such as intensity, duration, distribution and or timing of acceleration intensity over the day, or bouts duration [19, 23, 29].

Hence, the aim of this scoping review is to map advanced analytical approaches and their multidimensional summary variables used to provide a comprehensive picture of PA behaviour of an individual. Therefore, this scoping review will look at analytical methods that go beyond total PA volume, average daily acceleration and the conventional cut-point approaches, involving tri-axial accelerometer data from any sensor attachment location, and covering both data- and knowledge-driven techniques. We will also review the studies evaluating the association between the identified multi-dimensional summary variables with different health parameters. These methods may be used in future studies investigating the association between PA and health outcomes (e.g. cardio-metabolic health, diabetes, frailty), as well as in future personalised interventions in public health. This work will provide researchers as well as consumer wearable device companies with decisive information on future developments in the data processing, as well as on relevant feedback to the end user.

## Methods

### Protocol design

A scoping review will be conducted to map and collate the results of the available studies that address the research question. A scoping review is the most suitable approach for knowledge synthesis in those disciplines that are characterised by heterogeneity in methodological designs. In addition, scoping reviews also aim to identify and analyse knowledge gaps [30]. The analysis of accelerometer raw time series data is one such area of research where diverse methods have recently emerged with different purposes and constraints.

The framework of this scoping review will be guided by the Joanna Briggs Institute’s reviewer’s manual [31]. The present protocol is being reported in accordance with the Preferred Reporting Items for Systematic reviews and Meta-Analyses protocols (PRISMA-P) [32] (see checklist in Additional file 1), as suggested in the PRISMA Extension for Scoping Review (PRISMA-ScR) guidelines [33]. The scoping review will be conducted in accordance with the framework developed by Arksey and O’Malley [34].

The stages for the review will be the following:

1. Identifying the research question and objectives
2. Identifying relevant studies
3. Study selection
4. Data extraction and charting the data
5. Collating, summarising and reporting the data on analytical approaches, their multidimensional summary variables, as well as the association of health parameters with these variables

### Stage 1: Identification of the research question and objectives

The main purpose of undertaking this scoping review is to identify, collate and synthesise the results reported in the studies employing advanced analytical approaches to analyse PA behaviour. The principal research question guiding this scoping review is as follows:

- Which are the analytical approaches and their multidimensional summary variables used to provide a comprehensive picture of PA behaviour of an individual?

The secondary research questions are the following:

- What are the strengths and limitations of these analytical methods and their summary variables?
- Which of these summary variables have been studied for associating PA with health condition?

**Fig. 1:**
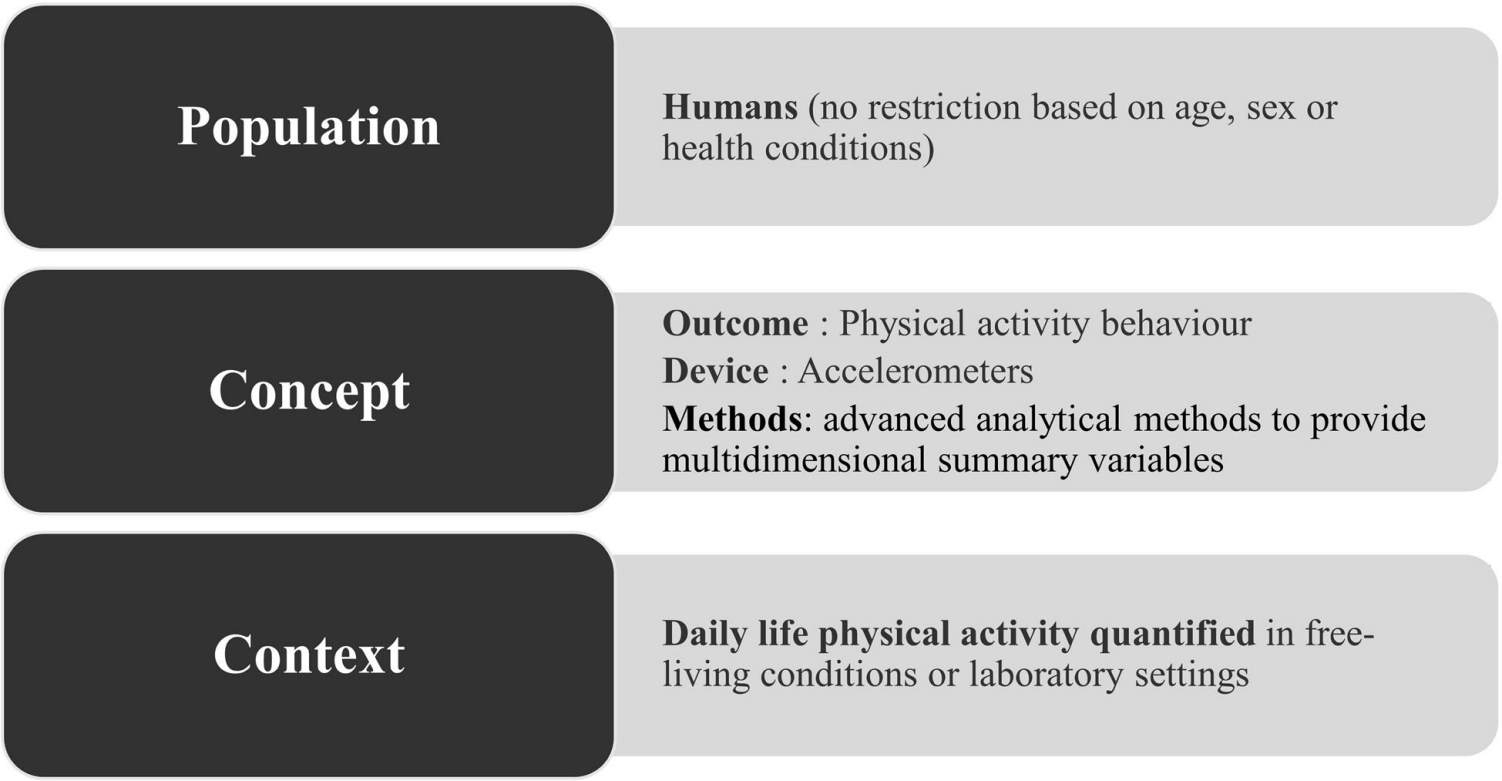
PCC framework

The study will use the population, concept and context (PCC) framework recommended by the Joanna Briggs Institute for Scoping Reviews. The PCC framework to determine our research questions and the search strategy is illustrated in Fig. 1. Key inclusion/exclusion criteria will be defined to support the search strategy.

### Stage 2: Identifying relevant studies

#### Data sources

MEDLINE (PubMed), Embase and Web of Science databases will be searched to find potentially relevant literature. Based on the PCC framework, a search strategy will be developed in Embase using an extensive list of synonyms for PA, accelerometers and outcome on the basis of keywords from important publications related to the topic. It will be prepared in consultation with the academic librarian of the Luxembourg Institute of Health. The list of synonyms will be used as keywords for the formulation of the search equation. To identify relevant literature, the keyword search will be restricted to the ‘title, abstract, and keywords’ fields. Emtree (Embase) and MeSh (MEDLINE) terms are not used to develop the search equation, because some of the important keywords are expanding into various other terms which are not relevant to our research aim. Web of Science does not have a comparable subject heading tool (thesauri). The search strategy will be adapted for each database. The search strategy was agreed on by TR, AB and LM, and the search will be conducted by TR and AB. The draft search strategy for Embase can be found in Additional file 2.

Further, the reference list of each included study and relevant reviews will be screened by the two reviewers (TR, AB) to find other relevant literature (backward search). A search in all the databases will be carried out to look for relevant literature that has cited the included studies (forward search).

Articles retrieved from the electronic databases will be downloaded in EndNote® (version 8). Endnote library will be used for the removal of duplicates and the sorting of the articles based on the inclusion and exclusion criteria. Endnote will also be used to manage full texts of the relevant literature to be included.

#### Eligibility criteria

**Fig. 2:**
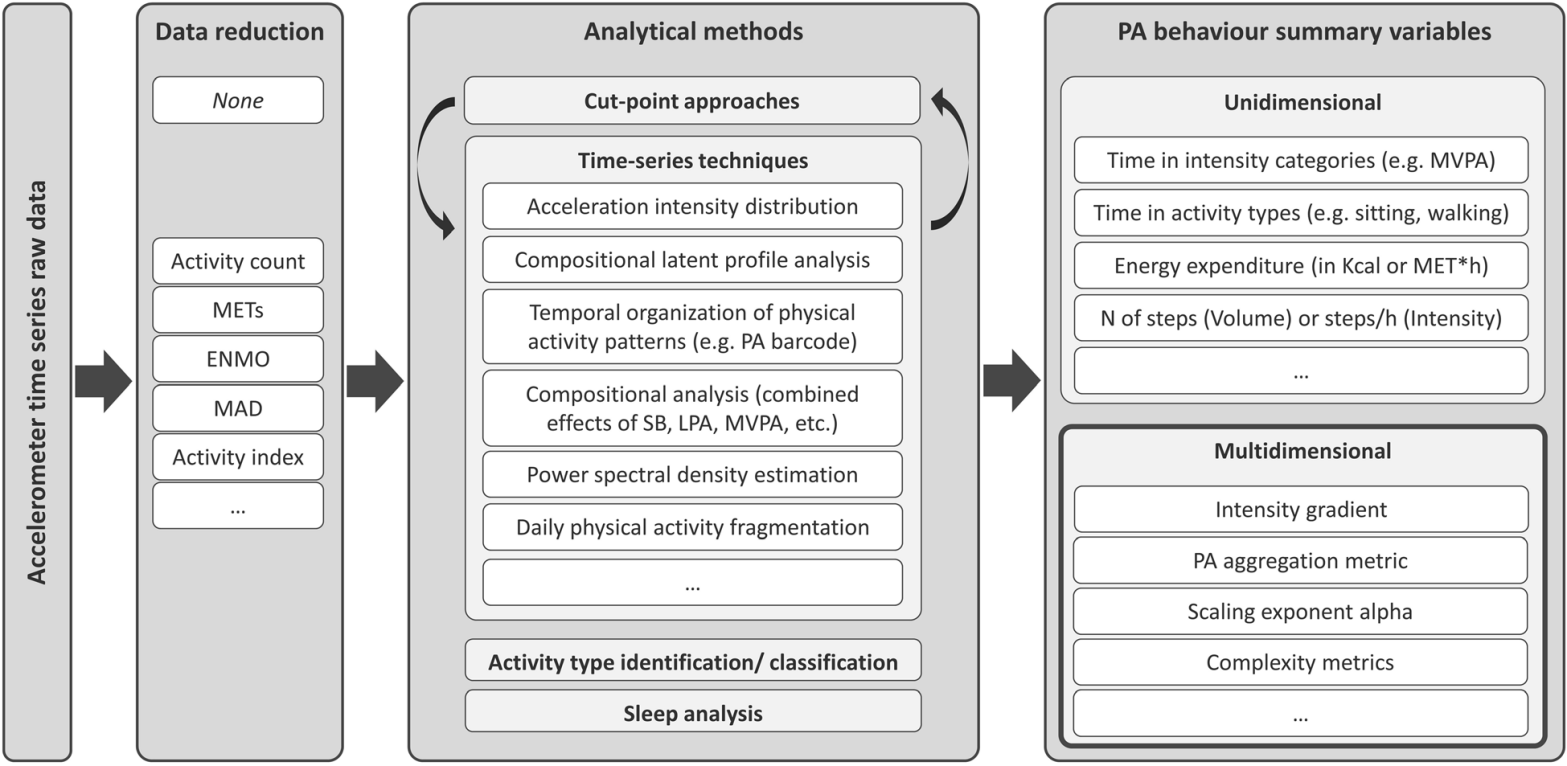
Conceptual framework of analytical methods for assessing PA behaviour using accelerometer raw time series data. The scoping review will specifically focus on approaches based on time series techniques applied to accelerometer raw data and provide multidimensional PA behaviour summary variables. PA, physical activity; MVPA, moderate to vigorous physical activity; LPA, light physical activity; SB, sedentary behaviour; MET, metabolic equivalent of task; MAD, mean amplitude deviation; ENMO, euclidean norm minus one

The PCC framework as illustrated in Fig. 1 will be used to define inclusion and exclusion criteria. Besides, a conceptual framework (Fig. 2), which presents a non-exhaustive list of already available approaches and summary variables, was developed by the research team to illustrate the motivation for the present review and to define the area of interest.

The selection of articles will be limited to studies published in English, including articles in press, from January 2010 onwards, considering we are interested in analytical methods that have been used in combination with accelerometer raw data, which have been developed since 2010 [14, 25, 35]. Studies including non-human subjects, grey literature, reviews and opinion articles will be excluded. However, reference lists from the reviews on related topics will be screened for additional potentially relevant articles. Studies identifying the type of PA through supervised classification algorithms (i.e. machine learning and artificial neural network approaches) will be excluded because systematic reviews were recently conducted on this topic [36, 37].

#### Inclusion criteria

1. Studies assessing PA behaviour
2. Studies gathering objective measurements of PA using research-grade or commercial-grade accelerometers
3. Studies using advanced analytical methods based on time series techniques
4. Studies computing multidimensional PA summary variables based on accelerometer raw time series data

#### Exclusion criteria

1. Studies using only unidimensional summary variables
2. Studies investigating sleep or sedentary behaviour as single standalone sub-domain of the physical behaviour
3. Studies focusing specifically on the identification of the type of PA through machine learning and artificial neural network approaches
4. Studies analysing a specific movement to investigate improvement in physical fitness or performance
5. Studies investigating methods combining raw data from accelerometer and other sensors such as thermometers, inclinometers, pulsometers or light intensity or skin conductance sensors
6. Articles such as commentary, editorial reviews and opinion articles
7. Studies not conducted in humans
8. Studies not published in English
9. Studies published before January 2010

### Stage 3: Study selection

The studies retrieved from abovementioned databases will be combined, and all the duplicates will be removed. A two-step process will be adopted for identifying the relevant studies. In the first step, two reviewers (TR, AB) will screen the titles and abstracts of the studies. Studies not meeting the eligibility criteria will be excluded. After screening of approximately 100 articles, the results will be compared to ensure consistency and resolve any incongruity. During this step, the detected protocols and reviews will also be screened for further potentially relevant articles. First, the abstracts will be screened. Then the reference lists of the remaining reviews will be checked for further articles. Similarly, potential studies that may result from relevant protocols will also be searched.

During the second step, the two authors (TR, AB) will be reading the full-text of the selected articles to check for the eligibility criteria. The reason for exclusion will be recorded. The articles meeting all the eligibility criteria will be used for data extraction. For each of the two steps, if there are disagreements between the reviewers, they will discuss the differences, and a third reviewer (LM) will be invited if they cannot reach a consensus.

### Stage 4: Data extraction and charting the data

In order to describe the different analytical methods and outputs, their meaning, strengths and limitations as well as their association with different health outcomes, the data from selected articles will be extracted by two reviewers (TR, AB) independently by means of a data extraction form. A draft of the data extraction form is available in Additional file 3. The data extraction form will be tested on five different studies to ensure the functionality of the form. Any disagreement will be resolved through discussion and if required discussion with the a third reviewer (LM). During the data extraction process, the researchers regularly compare the extracted data to ensure consistency. The data extraction form may be refined/adapted on the basis of this experience and inputs from the Competence Center for Methodology and Statistics (Luxembourg Institute of Health).

### Stage 5: Collating, summarising and reporting the data on analytical approaches, their multidimensional summary variables, as well as the association of health parameters with these variables

The data will be presented in the form of two tables. The first table will present the study designs, methodology and PA summary variables, whilst the second table will describe the association of the variables with the studied health outcomes. A synthesis of the results will describe the key characteristics of these studies and how these studies profiled PA and health behaviour. Further, the tables may be refined or the results may be presented in a graphical format based on the data extracted in order to present the results in a lucid and comprehensible manner. For example, Fig. 2 in the present protocol may be completed with the methods and multidimensional summary variables identified in the scoping review.

#### Quality assessment and risk of bias

The aim of the scoping review is to provide a global overview of available advanced analytical approaches used to assess a person’s PA profile, irrespective of study quality. The validity of the identified methods and variables is not relevant to our scoping review objectives. Quality assessment criteria and risk of bias would not have any impact on the eligibility criteria for study selection. Besides, in order to ensure the mapping of the highest possible number of present approaches, the inclusion should not be restricted by study quality. Hence, the quality of the identified literature will not be assessed.

## Discussion

The accuracy of a PA measurement as well as the dimensions covered by the outcomes depend on the analytical approaches and the outcome(s) calculated. It is important to select the appropriate outcomes depending on which dimensions of PA are important for a certain health condition. Through this scoping review, the authors look forward to systematically map the available cut-point independent and multidimensional variables, as well as to identify potential knowledge gaps on analytical methods for the assessment of PA. The results of this review may be used to guide future research related to the assessment of PA and the individualised feedback to each person. The results of this scoping review may be of interest to sports scientists, clinical researchers and smartphone application developers in the field of PA.

This scoping review will have some potential limitations. We will not discuss the accelerometer-based approaches that combine measures from other sensors measuring heart rate variability, temperature changes, oxygen saturation or skin conductance to name but a few. Besides, studies investigating sleep or sedentary behaviour as a single standalone sub-domain of the physical behaviour fall out of the scope of the present review. Studies identifying the type of PA through machine learning and artificial neural network approaches will not be included as this topic was covered by recent systematic reviews.

Ethical approval is not required for scoping reviews as it is based on the analysis of published data and results. Findings of this scoping review will be disseminated through peer-reviewed journals and conferences to contribute towards the development of PA measurement and health promotion in the community.

## Supplementary Information

**Additional file 1.** Preferred Reporting Items for Systematic reviews and Meta-Analyses Protocols (PRISMA-P) Checklist

**Additional file 2.** Draft search strategy for Embase.

**Additional file 3.** Draft of the data extraction form.

## Data Availability

All data generated or analysed during this study are included in this published article.

## Abbreviations

PA: Physical activity
MVPA: Moderate to vigorous physical activity
LPA: Light physical activity
SB: Sedentary behaviour
MET: Metabolic equivalent of task
MAD: Mean amplitude deviation
ENMO: Euclidean norm minus one
OSF: Open science framework
PRISMAP: Preferred Reporting Items for Systematic Review and Meta-Analysis Protocols
PRISMA-ScR: The Preferred Reporting Items for Systematic reviews and Meta-Analyses extension for Scoping Reviews
PCC: Population, Concept, and Context framework
