# Supplementary material for: Advanced analytical methods to assess physical activity behaviour using accelerometer raw time series data: a protocol for a scoping review
Source: Syst Rev. 2020 Nov 7;9:259. doi: 10.1186/s13643-020-01515-2 (PMC7648952; doi:10.1186/s13643-020-01515-2)
Supplement: Supplementary file 2 — Additional file 2. Draft search strategy for Embase. [file 13643_2020_1515_MOESM2_ESM.docx]

**Additional file 2: Draft search strategy for Embase.**

| - **Search strategy** |
| --- |
| - ('physical activity':ti,ab,kw OR 'physical activities':ti,ab,kw OR 'physical function':ti,ab,kw OR 'activity profile':ti,ab,kw OR 'activity profil*':ti,ab,kw OR 'activity pattern':ti,ab,kw OR 'activity patterns':ti,ab,kw OR 'activity behavior':ti,ab,kw OR 'activity behaviour':ti,ab,kw OR 'movement behavior':ti,ab,kw OR 'movement behaviour':ti,ab,kw OR 'physical behaviour':ti,ab,kw OR 'physical behavior':ti,ab,kw OR 'sedentary behavior':ti,ab,kw OR 'sedentary behaviour':ti,ab,kw OR 'sedentary time':ti,ab,kw OR 'daily function':ti,ab,kw OR 'habitual behavior':ti,ab,kw OR 'habitual behaviour':ti,ab,kw OR 'behavioral pattern':ti,ab,kw OR 'behavioural pattern':ti,ab,kw OR 'behavioral patterns':ti,ab,kw OR 'behavioural patterns':ti,ab,kw OR 'sedentary lifestyle':ti,ab,kw OR 'sedentary lifestyles':ti,ab,kw) - AND - ('acceleromet*':ti,ab,kw OR 'accelerometry':ti,ab,kw OR 'accelerometer':ti,ab,kw OR 'wearable sensor':ti,ab,kw OR 'wearable sensors':ti,ab,kw OR 'wearable device':ti,ab,kw OR 'wearable devices':ti,ab,kw OR 'activity tracker':ti,ab,kw OR 'activity track*':ti,ab,kw OR 'activity sensor':ti,ab,kw OR 'activity sensors':ti,ab,kw OR 'activity monitor':ti,ab,kw OR 'activity monitor*':ti,ab,kw OR 'smart device':ti,ab,kw OR 'smart devices':ti,ab,kw) - AND - ('raw' OR 'metric*' OR 'algorithm*' OR 'variable*' OR 'approach' OR 'accelerometer-derived' OR 'accelerometer derived' OR 'movement profil*' OR 'physical activity outcome*' OR 'pa outcomes' OR 'sedentary behavio*r outcome*' OR 'sb outcome') - AND - [english]/lim - AND - [humans]/lim - AND - [2010-2020]/py |
