# Supplementary material for: Advanced analytical methods to assess physical activity behaviour using accelerometer raw time series data: a protocol for a scoping review
Source: Syst Rev. 2020 Nov 7;9:259. doi: 10.1186/s13643-020-01515-2 (PMC7648952; doi:10.1186/s13643-020-01515-2)
Supplement: Supplementary file 3 — Additional file 3. Draft of the data extraction form. [file 13643_2020_1515_MOESM3_ESM.docx]

**Additional file 3: Draft of the data extraction form.**

| *Study characteristics*  Author and year  Description of the population  Age  Sample size  Study design  Details of the device used (e.g. type, wear- location, etc.)  Follow-up period (measurement length: days and hours per day)  Health condition  Health parameters / Biomarkers of disease  Conclusion of the study  *Analytical method description*  Name of the method  Description of the method  Limitations of the method  Strengths of the method  Software available to apply the analytical method to raw data (Yes/No)  If a software is available, is it open source (Yes/No)  Algorithms presented in the article (Yes/No)  *Association with health conditions*  Type of association  Details of association (e.g. linear association, significance, etc.)  Evidence on association between PA outcomes and health |
| --- |
